# Tobacco Nitrosamine Exposures Contribute to Fetal Alcohol Spectrum Disorder Associated Cerebellar Dysgenesis

**DOI:** 10.5539/ijb.v8n3p10

**Published:** 2016-07

**Authors:** Edward Re, Ming Tong, Suzanne M. de la Monte

**Affiliations:** 1 Department of Medicine, Division of Gastroenterology, and Liver Research Center Rhode Island Hospital, Providence, RI; 2 Departments of Pathology, Neurology, and Neurosurgery, and the Division of Neuropathology, Rhode Island Hospital, Providence, RI; 3 Warren Alpert Medical School of Brown University, Providence, RI

**Keywords:** fetal alcohol spectrum disorder, tobacco, cerebellum, NNK, nitrosamines

## Abstract

Variability in the phenotypic features and severity of fetal alcohol spectrum disorder (FASD) is not fully linked to alcohol dose. We hypothesize that FASD-type neurodevelopmental abnormalities may be caused by exposures to the tobacco-specific nitrosamine, NNK, since a high percentage of pregnant women who drink also smoke. In vitro experiments using PNET2 human cerebellar neuronal cultures examined ethanol and NNK effects on viability and mitochondrial function. Early postnatal rat cerebellar slice cultures were used to examine effects of ethanol and NNK on cerebellar histology and neuroglial and stress protein expression. Ethanol (50 mM) decreased viability and ATP content and increased mitochondrial mass, while NNK (100 μM or higher) selectively inhibited mitochondrial function. The slice culture studies demonstrated striking adverse effects of ethanol, NNK and ethanol+NNK exposures manifested by architectural disorganization of the cortex with relative reductions of internal granule cells, increases in external granule cells, and loss of Purkinje cells. Ethanol, NNK, and ethanol+NNK inhibited expression of choline acetyltransferase (ChAT) and acetylcholinesterase (AChE), and increased levels of 4-hydroxynonenal (HNE). In addition, ethanol increased activated Caspase 3, NNK decreased tau and phospho-tau, and ethanol+NNK inhibited expression of Aspartyl-β-hydroxylase (ASPH), which mediates neuronal migration. In conclusion, ethanol and NNK were shown to exert independent but overlapping adverse effects on cerebellar cortical development, neuronal viability, function, and neuroglial protein expression. These findings support our hypothesis that NNK exposures via tobacco smoking in pregnancy can contribute to FASD-associated neurodevelopmental abnormalities.

## 1. Introduction

### 1.1 Molecular Mediators of Neurodevelopmental Abnormalities in Fetal Alcohol Spectrum Disorder

Alcohol abuse during pregnancy causes fetal alcohol spectrum disorder (FASD) which leads to long-term neurodevelopmental deficits (Mattson, Crocker, & Nguyen, 2011; Riley, Infante, & Warren, 2011). Ethanol mediates its adverse effects on the immature brain by inhibiting insulin and insulin-like growth factor (IGF) signaling from ligand-receptor binding (de la Monte & Wands, 2010) to downstream pathways that promote neuronal growth, survival, metabolism, migration, and plasticity (Camp, Mayfield, McCracken, McCracken, & Alcantara, 2006; de la Monte et al., 2000; de la Monte, Neely, Cannon, & Wands, 2001; Hallak et al., 2001; Rajgopal & Vemuri, 2001; Zhang, Rubin, & Rooney, 1998).

### 1.2 Potential Cofactors in FASD Pathogenesis

Despite strong evidence that ethanol exposure is sufficient to cause FASD, in reality, humans who abuse alcohol, often also abuse tobacco products (Romberger & Grant, 2004). Review of an outpatient substance abuse treatment center database for pregnant women revealed that from 2010 to 2013, 74% of the pregnant alcohol users (N=57) smoked cigarettes compared with 42% of controls (N=31) (P=0.0053) (Tai et al., 2014). Although independent research has also demonstrated that cigarette smoking in pregnancy impairs fetal brain development (Jacobsen, Slotkin, Mencl, Frost, & Pugh, 2007), the mechanisms are not well understood. Given the high frequency of dual alcohol and tobacco exposures and their known independent teratogenic effects on the immature brain, further research is needed to assess potential co-factor effects of smoking in relation to FASD.

### 1.3 Mechanistic Supporting Data

First- and second-hand tobacco smoke exposures have long-term adverse effects on health due to co-exposures to highly toxic substances, including tobacco-specific nitrosamines such as 4-(methylnitrosamino)-1-(3-pyridyl)-1-butanone (NNK) and its metabolites (Duell, 2012; Go, Gukovskaya, & Pandol, 2005). Nitrosamines, including, those present in tobacco smoke, cause DNA damage and form adducts with proteins, lipids, and nucleic acids (Lao, Yu, Kassie, Villalta, & Hecht, 2007; Upadhyaya, Lindgren, & Hecht, 2009; Zabala et al., 2015). However, other effects related to brain development have not yet been investigated.

### 1.4 Hypothesis

Prenatal/early developmental exposure to NNK via tobacco smoke can separately and additively contribute to neurodevelopmental cerebellar abnormalities typically designated as features of FASD. Herein, we examined independent and additive effects of ethanol and NNK exposures on cell viability, mitochondrial function, and protein expression using the human PNET2 cerebellar neuronal line and primary rat cerebellar slice cultures. The cerebellum was studied because it is highly susceptible to teratogenic effects of prenatal alcohol exposure.

## 2. Methods

### 2.1 PNET2 Culture Studies of Ethanol and Graded NNK Effects on Neuronal Viability, Mitochondrial Mass, and Mitochondrial Function

Human cerebellar primitive neuroectodermal tumor 2 (PNET2) cells [The, et al] were maintained in Dulbecco’s modified Eagle’s medium (DMEM) supplemented with 5% fetal calf serum, 4 mM glutamine, 10 mM non-essential amino acid mixture (Gibco-BRL, Grand Island, NY), 25 mM KCl, and 9 g/L glucose (full media). Micro-cultures (96-well; 2x10^4^ cells/well) incubated at 37°C in sealed humidified chambers flushed with gas containing 75% nitrogen, 20% oxygen, and 5% carbon dioxide used throughout. Ethanol treatments were accomplished by adding 100 mM ethanol to both culture medium and the reservoir tray (Karl & Fisher, 1993). Sealed humidified chambers ensured a steady delivery of vaporized ethanol to the cultures. Control cultures had water added to the reservoir tray. The media and reservoir fluid were changed daily. At the time of seeding, control and ethanol-exposed cultures were treated with NNK (0–1 mM). Cultures were analyzed after 48 hours of treatment. Mitochondrial activity was measured with the 3-(4,5-dimethylthiazol-2-yl)-2,5-diphenyl tetrazolium bromide (MTT) assay (Sigma). Cell density, mitochondrial mass, mitochondrial oxidative phosphorylation, and adenosine triphosphate (ATP) content were measured by Hoechst H33348 staining, MitoTracker Green and MitoTracker Red fluorescence, and the ATPLite assay, respectively an according to the manufacturers’ and our previously published methods (Carter et al., 2008; de la Monte & Wands, 2002; Soscia et al., 2006; Xu et al., 2003).

### 2.2 Cerebellar Slice Cultures to Examine Independent and Additive Effects of Ethanol and NNK on Cerebellar Structure

Long Evans rat pups were administered intraperitoneal (i.p.) injections (50 μl) of saline or ethanol (2.5 g/kg in saline) on postnatal days (P) 3, 5, and 7(de Licona et al., 2009; Li et al., 2010; Silvers et al., 2006).. On P8, the rats were sacrificed and cerebella were harvested to generate slice cultures that were further exposed to ethanol (50 mM) and/or NNK (100 μM) for 72 hours (Le, Tong, Nguyen, & de la Monte, 2013). To generate slice cultures, cerebella were chilled in ice-cold full media (see above) supplemented with 120 IU/ml Penicillin and 120 μg/ml Streptomycin, and sliced at 250 μm intervals with a McIllwain tissue chopper (Mickle Laboratory Engineering Co. Ltd, UK). The slices were carefully separated under a dissecting microscope and placed into 8 μm pore inserts (BD Falcon) seated in 12-well Nunc plates. NNK (100 μM) or vehicle (0.1% DMSO) was added to each well. Cultures were maintained at 37°C in sealed humidified chambers as described earlier, except that 50 mM ethanol was used if cultures were generated from ethanol-exposed pups. At the time of harvest, cultured slices were divided for histology or biochemical studies. Cerebellar slices were fixed in 10% neutral buffered formalin and embedded in paraffin. Histological sections (5 μm-thick) were stained with Hematoxylin and eosin (H&E). Fresh tissue was snap frozen and stored at −80°C. The Lifespan-Rhode Island Hospital IACUC committee approved these procedures and the use of rats in experiments.

### 2.3 Duplex Enzyme-linked immunosorbent assays (ELISAs) to Assess Ethanol and NNK Effects on Neuronal, Injury, and Stress Associated Protein Expression

Direct binding duplex ELISAs measured immunoreactivity to tau, phospho- (p) tau (phosphorylated on S396 and T205), choline acetyltransferase (ChAT), acetylcholinesterase (AChE), aspartyl-β-hydroxylase (ASPH), 4-hydroxy-2-nonenal (HNE), and activated Caspase 3, with results were normalized to large acidic ribosomal protein (RPLPO) (Longato et al., 2012). Cerebellar slice culture tissue samples were homogenized in buffer containing 50 mM Tris (pH 7.5), 150 mM NaCl, 5 mM EDTA (pH 8.0), 50 mM NaF, 0.1% Triton X-100, and protease and phosphatase inhibitors (Le et al., 2013). Quadruplicate 50 μl aliquots containing 2 μg/ml of protein were adsorbed to the bottoms of 96-well MaxiSorp plates by overnight incubation at 4°C. Non-specific sites were adsorbed with Superblock-TBS. Proteins were reacted with primary antibody (0.1–0.4 μg/ml) overnight at 4°C. Immunoreactivity was detected with HRP-conjugated secondary antibody and the Amplex UltraRed soluble fluorophore. Protein homogenates were subsequently incubated with biotin-conjugated antibodies to RPLPO), and immunoreactivity was detected with streptavidin-conjugated alkaline phosphatase (1:1000) and the 4-Methylumbelliferyl phosphate (4-MUP) fluorophore. Fluorescence intensity was measured in a SpectraMax M5 microplate reader (Molecular Devices, Sunnyvale, CA) at Ex565nm/Em595nm for Amplex UltraRed, and Ex360nm/Em450nm for 4-MUP. Binding specificity was assessed with control incubations in which the primary or secondary antibody was omitted. The calculated ratios of immunoreactivity corresponding to the specific proteins/RPLPO were used for inter-group comparisons (Longato et al., 2012).

### 2.4 Statistical Analysis of Data

All assays were performed with 8 cultures per group. Graphs depict calculated mean ± S.D. of results. Inter-group comparisons were made by T-test analysis using the Holm-Sidak method, with alpha=5.000%, or two-way analysis of variance (ANOVA) with the Tukey post-hoc test (GraphPad Prism 6, San Diego, CA).

### 2.5 Materials Used in Experiments

Pharmaceutical grade ethanol was used in all experiments. The A85G6 and A85E6 monoclonal antibodies to ASPH were generated to human recombinant protein (de la Monte et al., 2009) and purified over Protein G columns (Healthcare, Piscataway, NJ). Otherwise, antibodies used for duplex ELISAs were purchased from Abcam (Cambridge, MA). RPLPO) antibody was from the Proteintech Group Inc (Chicago, IL). ELISA MaxiSorp 96-well plates were purchased from Nunc (Rochester, NY). Horseradish peroxidase (HRP)-conjugated secondary antibody, Amplex Red soluble fluorophore, and the Akt Pathway Total and Phospho panels were purchased from Invitrogen (Carlsbad, CA). HRP-labeled polymer conjugated secondary antibody was purchased from Dako Corp (Carpinteria, CA). The SpectraMax M5 microplate reader was purchased from Molecular Devices Corp. (Sunnyvale, CA). BCA reagents were from Pierce Chemical Corp. (Rockford, IL). All other fine chemicals, including NNK were purchased from CalBiochem (Carlsbad, CA), Pierce (Rockford, IL), or Sigma (St. Louis, MO).

## 3. Results

### 3.1 Ethanol and NNK Effects on PNET2 Neuronal Viability and Mitochondrial Function

**Figure 1:**
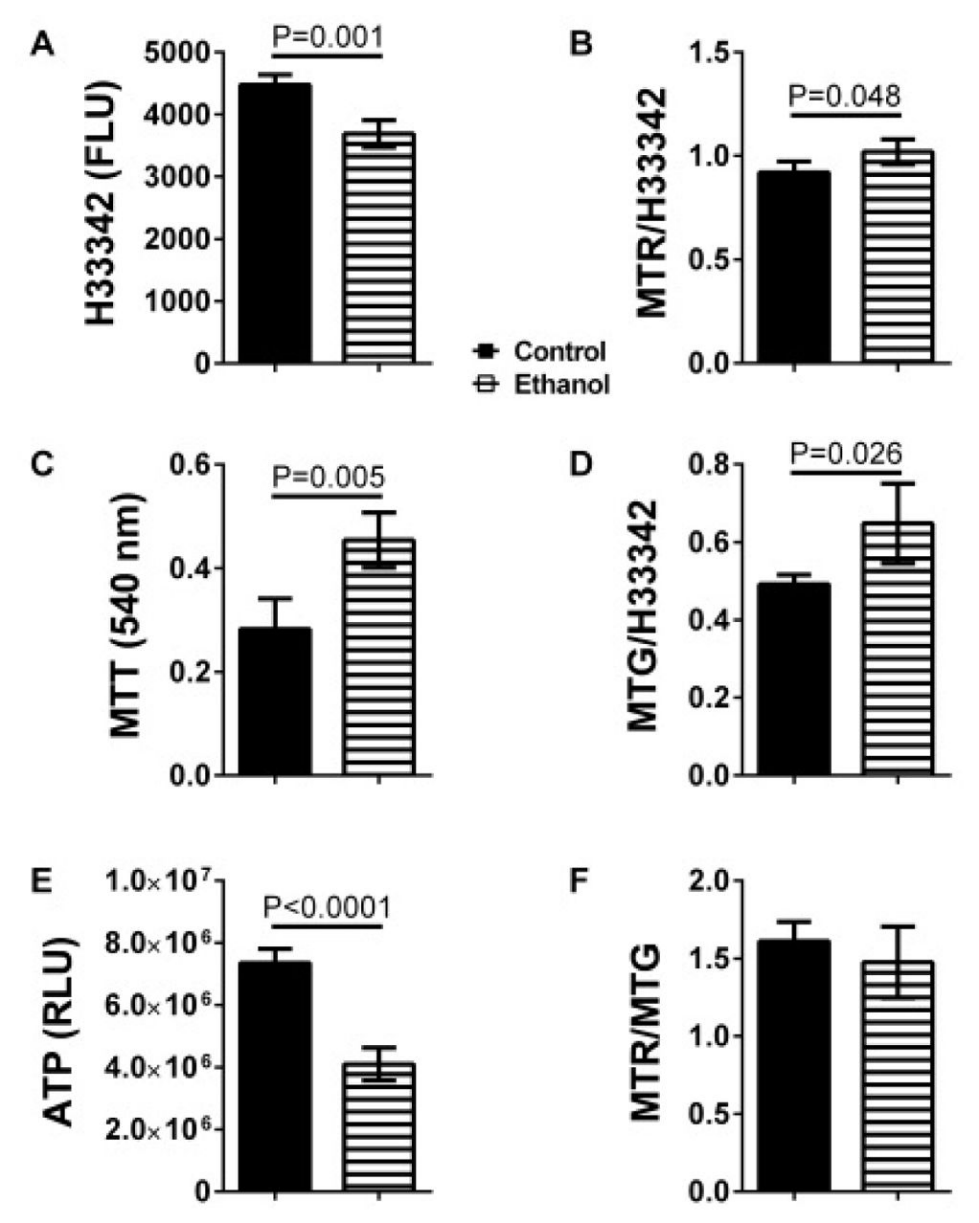
Effects of ethanol on neuronal viability, mitochondrial mass, and mitochondrial function. Human PNET2 cultures seeded into 96-well plates were exposed to 0 or 100 mM ethanol for 48 hours. (A) H33342 fluorescence, (B) MitoTracker Red (MTR) fluorescence, (C) MTT activity, (D) MitoTracker Green (MTG) fluorescence, and (E) ATP content were measured as indices of cell number (viability), mitochondrial oxidative phosphorylation, mitochondrial activity, mitochondrial mass/proliferation, and ATP production, respectively (see Methods). (F) MTR/MTG ratios were calculated to assess relative mitochondrial function/mitochondrial mass. 8 replicate cultures were assayed per group. Data points reflect Mean ± S.D. of results. Inter-group comparisons were made with unpaired, two-tailed Student T-tests. Significant differences are indicated in the panels

Ethanol significantly reduced H33342 fluorescence, reflecting cell number/culture viability (Figure 1A), and ATP content (Figure 1E), and increased MTT activity (Figure 1C), MitoTracker Red (MTR; Figure 1B), and MitoTracker Green (MTG; Figure 1D) fluorescence, reflecting mitochondrial activity, oxidative phosphorylation, and mass respectively. However, similar MTR/MTG ratios, reflecting mitochondrial activity relative to mass, were observed in control and ethanol-exposed cultures (Figure 1F).

**Figure 2:**
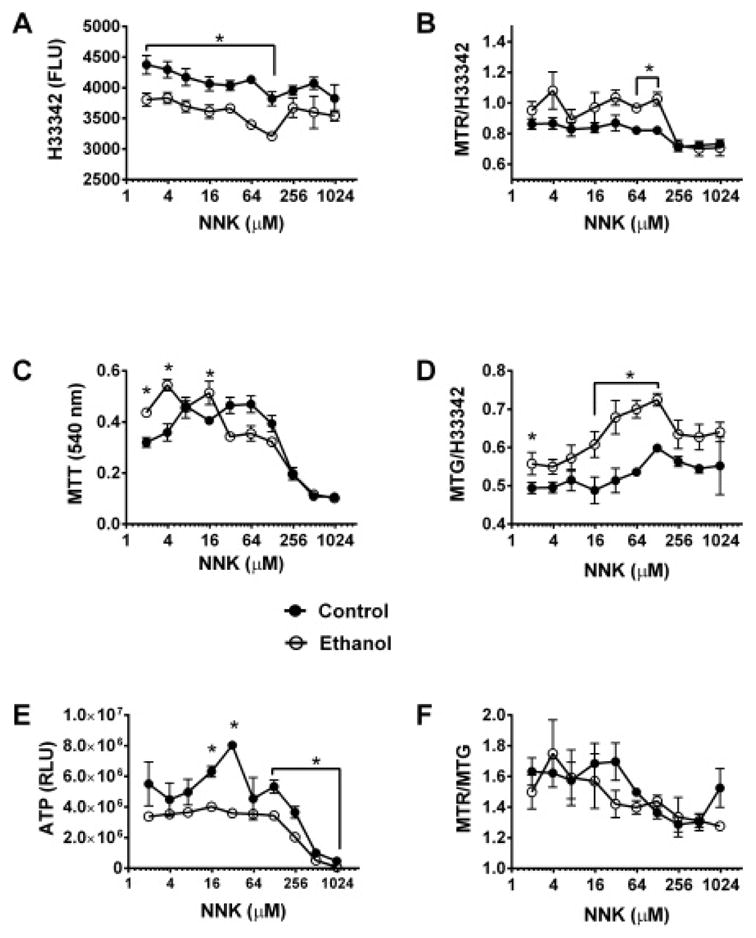
NNK dose effects on neuronal viability, mitochondrial mass, and mitochondrial function. Control and ethanol exposed human PNET2 cerebellar neuronal 96-well cultures were exposed to 0 or 100 mM ethanol plus 0.5–1000 μM NNK for 48 hours. (A) H33342 fluorescence, (B) MitoTracker Red (MTR) fluorescence, (C) MTT activity, (D) MitoTracker Green (MTG) fluorescence, and (E) ATP content were measured as indices of cell number (viability), mitochondrial oxidative phosphorylation, mitochondrial activity, mitochondrial mass/proliferation, and ATP production, respectively (see Methods). (F) MTR/MTG ratios were calculated to assess relative mitochondrial function/mitochondrial mass. 8 replicate cultures were assayed per time point, per group. Data points reflect Mean ± S.D. of results. Inter-group comparisons were made using the Holm-Sidak T-test with alpha=5%. Asterisks (*) indicate significant differences at specific NNK doses (P<0.005–0.0001)

Figure 2 illustrates NNK dose effects on H33342, MTT, ATP, MTR, MTG, and MTR/MTG in control and ethanol-exposed cultures. In both control and ethanol-exposed cultures, H33342 fluorescence declined just slightly with increasing NNK dose (Figure 2A). Although H33342 fluorescence was consistently, and for the most part (up to 100 μM NNK) significantly lower in the ethanol relative to control cultures, the gap was not enhanced by NNK exposure, suggesting that NNK had no additional negative impact on neuronal viability. In contrast, NNK had striking and similar dose effects on mitochondrial function in control and ethanol-exposed cultures. Below 100 μM, the main effects of NNK on MTR (Figure 2B), MTT (Figure 2C), and ATP (Figure 2E) were due to ethanol as the relative differences were nearly the same as in vehicle-treated cultures. However, above 100 μM NNK, the mean levels of MTT and ATP declined sharply. MTR also declined sharply in control cultures at high doses of NNK. These effects resulted in similarly low levels of MTT, ATP, and MTR in control and ethanol-exposed cultures, indicating independent NNK effects on mitochondrial function. We also noted that at intermediate NNK doses, ATP was selectively increased in control cultures, while MTG was increased in ethanol-exposed cultures. MTG, which reflects mitochondrial mass, did not decline with increasing NNK dose (Figure 2D). The MTR/MTG ratios declined gradually and similarly in control and ethanol-exposed cultures such that none of the inter-group differences were statistically significant (Figure 2F).

### 3.2 Ethanol and NNK Effects on Cerebellar Development in Slice Cultures

**Figure 3:**
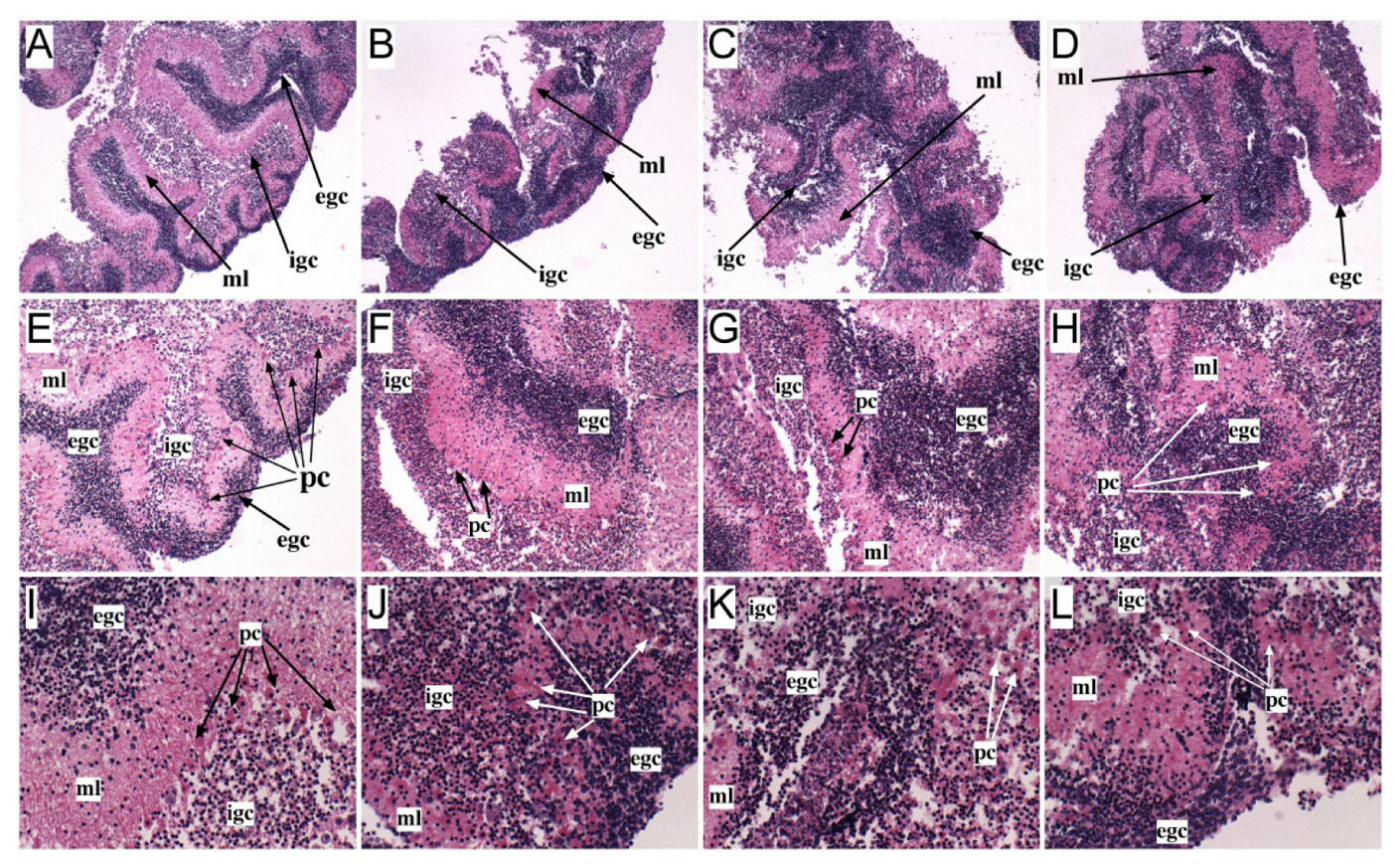
Teratogenic effects of ethanol, NNK and ethanol+NNK on rat cerebellar cortex. Cerebellar slice cultures were generated from Long Evans rat pups treated by i.p. injection of saline (control) or ethanol (2.5 g/Kg) on postnatal days 3, 5, and 7. Slice cultures were further treated with 100 μM NNK or vehicle (0.1% DMSO), and maintained in humidified chambers with water or 50 mM ethanol vaporized from the reservoir tray. In vitro ethanol exposure was used for cultures generated from ethanol-treated pups. Cultures were harvested after 72 hours. Formalin-fixed, paraffin-embedded histological sections were stained with H&E. (A, E, I) Control cultures-Note well-organized cortical architecture with complex fissuring (**-deep; * shallow), clearly delineated external granule cell (egc), molecular (ml), and internal granule cell (igc) layers, and abundant viable Purkinje cells (pc). (B, F, J) Ethanol, (C, G, K) NNK, and (D, G, L) ethanol+NNK exposures caused severe cerebellar hypoplasia (smaller size) and hypotrophy (reduced cell density) with irregular fissuring and disorganization of the larminar architecture (compare with control images), and relative increases in egc compared with igc. Ethanol-exposed cultures exhibited marked reductions in PC populations (3J), while NNK and ethanol+NNK exposed cultures showed modest reductions in PC but strikingly increased eosinophilic degeneration (3K, 3L). Original magnifications: 4x, A–D; 10x, E–H; 20x, I–L

Histological sections of control cerebellar slice cultures revealed intact cortical architectures with complex foliation (folding), including deep and shallow fissures (grooves) and clearly delineated lamination of external and internal granule cell, Purkinje, and molecular layers (Figures 3A, 3E). In addition, Purkinje cells were abundant and cytologically intact Figure 3I). Ethanol caused severe cerebellar (hypotrophy-small size) hypoplasia (reduced cell number) with disorganization of the normal cortical architecture (Figures 3B, 3F), increased thickness of the external and reduced thickness of internal granule layers (Figure 3F), and loss or eosinophilic degeneration (red cytoplasm, nuclear pyknosis corresponding to necrosis) of Purkinje cells (Figure 3J). NNK (Figures 3C, 3G, 3K) and ethanol+NNK (Figures 3D, 3H, 3L) exposure had similar effects in that both caused cerebellar hypotrophy and hypoplasia that were less severe than with ethanol, but striking disorganization of the cortical architecture, rendering it scarcely recognizable as cerebellum. The conspicuously greater abundances of external versus internal granule cell populations correspond with effects of impaired neuronal migration. Irregular foliation (fissuring) present in ethanol, NNK, and ethanol+NNK exposed cultures reflects significant abnormalities in morphogenesis. In both NNK and ethanol+NNK exposed cultures, Purkinje cells were abundant but exhibited prominent eosinophilic degeneration.

### 3.3 Neuroglial and Stress Proteins Modulated by Ethanol and/or NNK Exposure in Cerebellar Slice Cultures

Two-way ANOVA tests demonstrated significant ethanol effects on tau, ChAT, AChE, HNE, and activated Caspase 3 expression and a trend effect on pTau and ASPH (A85E6), corresponding to Humbug, i.e. catalytically inactive ASPH (Table 1). NNK exposures had significant effects on all proteins examined except ASPH (A85E6). Ethanol x NNK interactive effects were detected for tau, ChAT, AChE, ASPH (A85G6; catalytically active), and HNE.

**Figure 4:**
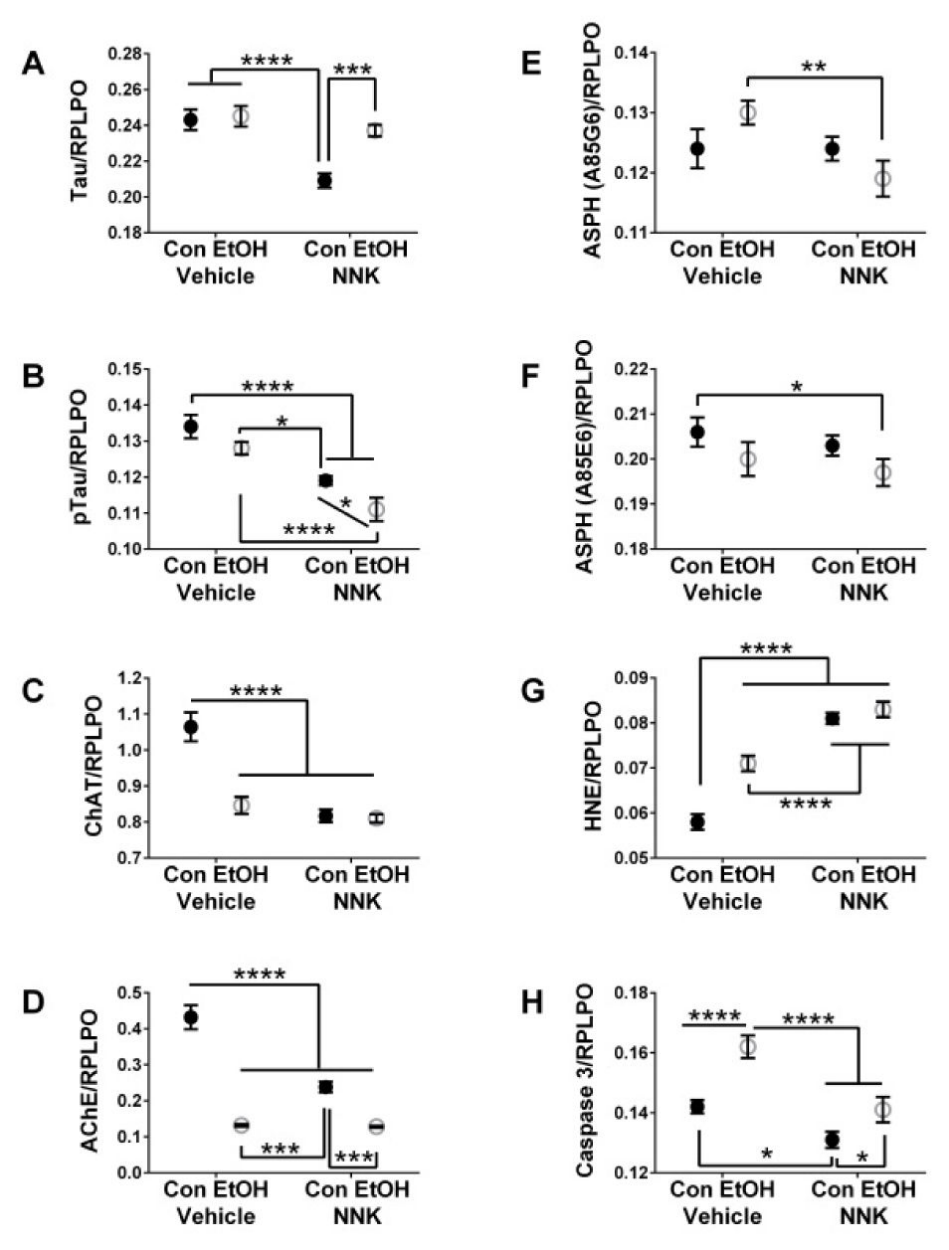
Ethanol, NNK, and ethanol+NNK modulation of neuronal and stress protein expression. Duplex ELISAs tandemly measured immunoreactivity to (A) Tau, (B) pTau, (C) choline acetyltransferase (ChAT), (D) acetylcholinesterase (AChE), (E) ASPH-aspartate-β-hydroxylase (ASPH)-A85G6, (F) ASPH-A85E6, G) 4-hydroxy-2-nonenal (HNE), and (H) activated Caspase 3, followed by RPLPO as an internal control for normalization of results (see Methods). Graphs depict mean ± S.D. of immunoreactivity measured in each group. Inter-group comparisons were made by two-way ANOVA (Table 1) with the post-hoc Tukey multiple comparison test. (*P<0.01; **P<0.01; ***P<0.001; ****P<0.0001)

Tau expression was selectively reduced by NNK relative to all other groups (Figure 4A), whereas pTau (phosphorylated on S396 and T205) expression was progressively reduced by ethanol, followed by NNK, and then further by ethanol+NNK exposures (Figure 4B). ChAT (Figure 4C) and AChE (Figure 4D) expression were significantly reduced by ethanol, NNK, and ethanol+NNK exposures relative to control. The mean levels of ASPH-A85G6 (Figure 4E) and ASPH-A85E6 (Figure 4F) were lowest in the ethanol+NNK cultures, and significantly differed from either the ethanol (Figure 4E) or control (Figure 4F) group. HNE immunoreactivity, a marker of lipid peroxidation, was significantly increased by ethanol, NNK, and ethanol+NNK exposures (Figure 4G). NNK and ethanol+NNK increased HNE levels significantly above those associated with ethanol-only treatment. Finally, ethanol selectively increased activated Caspase 3 immunoreactivity relative to the other three groups, while NNK-only treatment was associated with reduced levels of Caspase 3 expression (Figure 4H).

## 4. Discussion

Despite clinical and experimental evidence that ethanol exposure is sufficient to cause FASD, concerns about potential roles of co-factors such as tobacco smoke exposure were raised because: 1) the FASD phenotype is quite varied and its severity is not entirely linked to ethanol dose; and 2) a very high percentage of pregnant women who drink also smoke. We were led to examine independent and co-factor effects of NNK and ethanol during development because NNK is a tobacco-specific nitrosamine that has broad inhibitory effects on brain structure and function in adolescent and adult brains (Tong, Yu, Deochand, & de la Monte, 2015; Tong, Yu, Silbermann, et al., 2015), and other nitrosamines including N-nitrosodiethylamine (NDEA) and streptozotocin were previously shown to have teratogenic effects on the immature brain, including cerebellum (Andreani, Tong, & de la Monte, 2014; Lester-Coll et al., 2006; Tong, Longato, & de la Monte, 2010).

From the PNET2 culture experiments, we determined that ethanol and NNK had distinct adverse effects on neuronal cells. Ethanol decreased cell number and energy metabolism while increasing mitochondrial activity (MTR), mitochondrial proliferation (MTG), and MTT activity, consistent with previous reports (de la Monte et al., 2001; de la Monte & Wands, 2001). MTT activity, which measures NADPH-dependent oxidoreductase enzyme activity, carries complex significance because it can report cytotoxicity, cytostatic activity, or proliferation. The main effects of NNK occurred at the higher doses tested, i.e. 100 μM and above, which resulted in sharp declines in MTT, ATP, and MTR, and progressive declines in MTR/MTG. In contrast, cell number and mitochondrial mass (proliferation) were modestly affected. These findings indicate that the higher doses of NNK cause mitochondrial failure, irrespective of ethanol exposure. The absence of significant cell loss could represent an artefact of the short culture duration (48 hours). The lack of additive ethanol+NNK effects indicates that NNK’s adverse effects on mitochondrial function were dominant and independent of ethanol.

The slice culture experiments were performed with third-trimester equivalent cerebella from control and ethanol-exposed rat pups in which the NNK treatments were performed in vitro. The third-trimester equivalent exposures were used to target the most vulnerable period of cerebellar development in rats (Karacay, Li, & Bonthius, 2008). The results indicate that ethanol, NNK and ethanol+NNK had profound adverse effects on cerebellar growth and structure. The smaller sizes corresponded to hypotrophy, and reduced populations of Purkinje and internal granule cells reflect combined effects of hypoplasia and apoptosis or necrosis. The disorganized cortical architecture observed in all 3 experimental groups indicates that ethanol and NNK disrupt functions responsible for folding and neuronal migration. Expression levels of ASPH-A85G6 and ASPH-A85E6 were previously shown to be reduced cerebella following prenatal alcohol exposure (Carter et al., 2008; de la Monte et al., 2009). ASPH has demonstrated roles in neuronal migration and cell adhesion (Cantarini et al., 2006; Ince, de la Monte, & Wands, 2000; Lahousse, Carter, Xu, Wands, & de la Monte, 2006; Lawton, Tong, Gundogan, Wands, & de la Monte, 2010; Longato et al., 2009; Maeda et al., 2003) The reduced levels of ASPH-A85G6 and ASPH-A85E6 in ethanol+NNK exposed suggests that inhibition of ASPH may have contributed to the architectural disarray of the cerebellum. However, other factors were likely involved similar abnormalities occurred in the ethanol and NNK exposure groups. Other potential mediators could be mitochondrial dysfunction and increased oxidative stress (increased HNE) since ethanol, NNK, and combined exposures produced similar responses.

NNK selectively inhibited Tau expression, and NNK and ethanol+NNK had greater inhibitory effects pTau than did ethanol. Tau is an important component of the neuronal cytoskeleton and decreased levels of tau could contribute to cytoskeletal collapse and loss of synaptic connections. Tau phosphorylation mediates translocation of the neuronal cytoskeleton into neurites for establishing and maintaining synaptic connections and enabling axons to course through white matter. Therefore, after only a brief period of exposure, NNK has significant adverse effects on neuronal cytoskeletal structure and function, and the combined exposures further reduced Tau phosphorylation and probably also its function. The latter also suggests that the effects of ethanol and NNK were additive with respect to their inhibitory effects on tau phosphorylation.

Ethanol, NNK and ethanol+NNK exposures impaired cholinergic mechanisms as was manifested by the significant reductions in ChAT and AChE expression. Similar effects of in vivo or in vitro ethanol exposure have been reported previously (Cohen, Tong, Wands, & de la Monte, 2007; de la Monte et al., 2008; Jamal et al., 2009; Kuriyama, Ohkuma, Tomono, & Hirouchi, 1987; Le et al., 2013; Soscia et al., 2006), but the findings with respect to NNK during development are novel. Reduced ChAT is associated with decreased neurotransmission required for cerebellar motor function, and reduced expression of AChE negatively impacts neurotransmission due to impaired cell viability and function associated with persistent oxidative stress (Ansari, Abdul, Joshi, Opii, & Butterfield, 2009; dos Santos et al., 2011; Ehrich, Van Tassell, Li, Zhou, & Kepley, 2011; Kazi & Oommen, 2012).

HNE, a marker of lipid peroxidation, was significantly elevated in cerebellar slice cultures from all treatment groups relative to control. Lipid peroxidation has dire effects on membrane integrity, myelin maintenance, and cellular stress, and ultimately leads to cell death. The elevated HNE levels in ethanol, NNK, and ethanol+NNK treated cerebellar cultures suggest that lipid adduct formation is an important cause of oxidative stress in developing brains exposed to tobacco smoke. The higher levels of HNE in NNK- versus ethanol-treated cultures further suggest that lipid peroxidation adducts may accumulate more effectively with smoking than drinking.

Corresponding with the inhibitory effects of ethanol and not NNK on culture viability demonstrate in PNET2 cells, activated Caspase 3 expression was increased only in the ethanol-exposed cerebellar slice cultures. This suggests that ethanol-associated cerebellar hypoplasia was mediated by combined effects of increased apoptosis and oxidative stress (via lipid peroxidation), whereas NNK mainly promoted oxidative stress. The PNET2 cerebellar neuron culture results further suggest that mitochondrial dysfunction is a source of oxidative stress in both ethanol- and NNK-exposed cultures.

In conclusion, these studies demonstrate that developmental exposures to ethanol or NNK adversely affect cerebellar structure and function; with responses ranging from overlapping to distinct. Studies in human PNET2 cells suggest that ethanol impairs neuronal viability and mitochondrial function, whereas NNK mainly impairs mitochondrial function. The slice culture studies demonstrated striking ethanol-, NNK-, and ethanol+NNK-induced cerebellar architectural abnormalities associated with deficits in ChAT and AChE and increased levels of HNE (lipid peroxidation). A distinct effect of ethanol was to increase activated Caspase 3, suggesting that cell loss was mediated by apoptosis. NNK’s adverse effects on tau and pTau reflect abnormalities that could contribute to collapse of the neuronal cytoskeleton and synaptic disconnection. Finally, additive effects of ethanol and NNK occurred with respect to pTau and ASPH (A85G6 and A85E6), suggesting that impairments in neuronal migration and structure were more adversely affected by the dual exposures compared with either one alone. Altogether, the findings support the hypothesis that exposure to NNK via tobacco smoke can contribute to brain abnormalities that characteristically occur in FASD.

## Figures and Tables

**Table 1:** Two-way ANOVA summary of ethanol and NNK effects on protein expression in cerebellar slice cultures-duplex ELISA results

Protein	Ethanol Effect		NNK Effect		Ethanol x NNK Effect	

	F-Ratio	P-value	F-Ratio	P-Value	F-Ratio	P-Value
Tau	9.710	0.003	19.03	<0.0001	7.293	0.009
pTau	7.612	0.077	39.77	<0.0001	0.155	N.S.
ChAT	19.28	<0.0001	30.5	<0.0001	16.96	0.0001
AChE	124.5	<0.0001	29.03	<0.0001	26.73	<0.0001
ASPH (A85G6)	0.036	N.S.	4.390	0.040	4.390	0.040
ASPH (A85E6)	3.722	*0.058*	0.931	N.S.	0.000	N.S.
4-HNE	20.93	<0.0001	114.0	<0.0001	11.26	0.0014
Caspase 3	20.11	<0.0001	22.88	<0.0001	2.235	N.S.

Immunoreactivity was measured by duplex ELISAs in which results were normalized to large ribosomal protein measured in the same wells. Results were analyzed by 2-way ANOVA and the Tukey multiple comparisons post-test. F-ratios and P-values reflect variances with respect to Control vs Ethanol diets, Vehicle vs NNK treatments, and interactions between ethanol and NNK. Results are graphed in Figure 4. See text for abbreviations.
